# Cytoprotective roles for autophagy

**DOI:** 10.1016/j.ceb.2009.12.002

**Published:** 2010-04

**Authors:** Kevin Moreau, Shouqing Luo, David C Rubinsztein

**Affiliations:** Department of Medical Genetics, Cambridge Institute for Medical Research, University of Cambridge, Addenbrookes Hospital, Hills Rd, Cambridge, UK

## Abstract

Macroautophagy (referred to as autophagy in this review) is a genetically regulated bulk degradation program conserved from yeast to humans, in which cytoplasmic substrates, such as damaged organelles and long-lived proteins, are delivered to lysosomes for degradation. In this review, we consider recent data that highlight possible mechanisms whereby autophagy mediates cytoprotective effects. These include the ability of autophagy to buffer against starvation, protect against apoptotic insults and clear mitochondria, aggregate-prone proteins and pathogens. These effects are pertinent to the roles of autophagy in normal human physiology, including the early neonatal period and ageing, as well as a variety of diseases, including cancer, neurodegenerative conditions and infectious diseases.

## Introduction

Autophagy involves the formation of double-layered membrane structures around a portion of cytosol [1]. These membrane structures, called autophagosomes, are trafficked to lysosomes in a dynein-dependent manner along microtubules. The fusion of the outer membrane of the autophagome and the lysosome leads to the formation of autolysosome, where the contents are degraded. The identification of autophagy genes (*Atg* genes) in yeast heralded the start of a new era for autophagy research, as it allowed the identification of mammalian orthologues of these genes and many studies that have elucidated critical roles for this process in normal physiology and disease [2].

Autophagy occurs at basal, constitutive levels and is upregulated under physiological stress, for example, nutrient deprivation and growth factor withdrawal. Autophagy is essential in maintaining homeostasis, which requires protein degradation for energy needs, by removing damaged substrates for recycling. As a result, autophagy is important in promoting cell survival in different conditions, such as protein aggregate-induced stress, nutrient and growth factor deprivation, ER stress and pathogen infection [3]. The demand on autophagy differs among different cell types. It plays particularly important roles in non-dividing cells, for example, neurons and myocytes [3].

## Cytoprotective effects of autophagy

The occurrence of increased autophagosomes in dying/dead cells in certain contexts led to the idea that autophagy may be a primary death mediator. This phenomenon was termed type-II cell death, to differentiate it from apoptotic (or type-I cell death) [4]. Since increased numbers of autophagosomes can result both from increased autophagosome synthesis, as well as from decreased autophagosome–lysosome fusion, cells showing type-II cell death may not always have increased autophagic flux. Although there is mounting evidence that this may be a relevant pathway in *Drosophila* metamorphosis [5], the role of autophagy as a positive mediator of cell death is unclear and not well understood in mammalian systems. On the other hand, many studies suggest that impaired autophagy sensitises cells and organisms to toxic insults [6,7].

One of the first studies that addressed this question in mammalian cells [8] reported that cells exhibit type-II cell death morphology when lysosome–autophagosome fusion is inhibited, suggesting that autophagy may be cytoprotective. The lysosomotropic agent hydroxychloroquine (HCQ) leads to the accumulation of autophagic vacuoles (AV) that triggers a pre-lethal program, which can be suppressed by mitochondrial membrane permeability inhibitors, or caspase antagonists. AV accumulation precedes ΔΨ_*m*_ loss, which marks imminent cell death and apoptosis. Consistent with these data, Ravikumar *et al*. [9] showed that genetic or chemical inhibition of autophagy sensitised cells to pro-apoptotic insults and that autophagy induction with rapamycin or Beclin 1 overexpression protected cells and *Drosophila* from pro-apoptotic agents. These data suggested that the ability of autophagy to reduce the mitochondrial load in cells and thereby lower the amount of pro-apoptotic molecules (like cytochrome *c*) released from mitochondria after apoptotic insults, was an important contributor to the protective effects of autophagy.

## Autophagy alleviates starvation-induced stress

One of the archetypal roles of autophagy is to enable cell survival during starvation conditions. In unicellular organisms such as yeast and protozoans, cells with knockouts of autophagy genes die shortly after starvation induction, while they are viable in normal nutrient conditions [7]. This scenario has parallels in mammalian cells. IL-3 is essential for survival and growth of bone marrow cells and IL-3 deprivation induces cell death in these cells. IL-3 withdrawal results in progressive atrophy of Bax/Bak^−/−^ (apoptosis-incompetent) cells generated from bone marrow and increases autophagy, and these IL-3-dependent Bax/Bak^−/−^ cells undergo autophagy to maintain cell survival for over 20 weeks, while the inhibition of autophagy leads to cell death. The addition of IL-3 re-activates the catabolic process that maintains cell survival [10]. Although cell death will occur inevitably in persistent starvation conditions, this process can be greatly delayed in autophagy-competent cells, which survive by stopping growth and cell division. Conversely, autophagy inhibition sensitises cells to nutrient starvation-induced cell death [8].

Currently, it is widely accepted that autophagy is a self-limited survival strategy, rather than a primary or irreversible death trigger. Indeed, autophagy induction may also translate into a longevity mediator in a wide-range of organisms ([11^•^] and references therein).

## Mechanisms of autophagy-mediated survival

The mechanisms whereby autophagy mediates its cytoprotective effects are still the subject of active investigation by a number of laboratories. It is likely that these may vary according to the stress that the relevant cells and organisms are exposed to, and the cell types involved. In the rest of this review, we will consider these different mechanisms.

## Nutrient and bioenergetic homeostasis

By promoting catabolic reactions, autophagic degradation of proteins, organelles, membranes and lipid droplets [12^••^] generates amino acids and fatty acids and other new metabolic substrates, which provide building blocks and maintain bioenergetics for protein synthesis and ATP energy production. These events are particularly important for cells deprived of nutrients.

Autophagy is not only essential for cell survival but also essential for organismal survival when nutrients are scarce. An important period where this occurs in mammals is in the early neonatal period, before breastfeeding is established [13,14]. Mice lacking *Atg5* or *Atg7*, which are essential for autophagosome formation, die during this neonatal stage. Indeed, if neonatal mice are prevented from breastfeeding, the survival time of starved autophagy-deficient neonates is much shorter than that of wild-type counterparts. Conversely, the survival of the autophagy-deficient mice can be extended if they are hand-fed. These studies showed that autophagy was required for the liberation of energy during this period of nutrient deprivation.

## Removal of misfolded and aggregate-prone proteins

During aging, cells accumulate increasing amounts of misfolded cytoplasmic proteins, which frequently form various types of aggregates. This phenomenon is accentuated in a range of neurodegenerative diseases, including Alzheimer's diseases, Parkinson's diseases and Huntington's diseases. One of the roles of autophagy is to limit the accumulation of aggregate-prone intracytoplasmic proteins [15]. If autophagy is blocked, such proteins accumulate. Conversely, if autophagy is induced, the clearance of such proteins is enhanced and their toxicities are attenuated in cell, *Drosophila*, zebrafish and mouse models [16–19,20^••^].

When autophagy is compromised in normal mice, one also sees the accumulation of inclusions that are ubiquitinated [14,21,22^••^,23^••^]. Subsequent work showed that these inclusions are also decorated with antibodies against a ubiquitin-binding protein, called p62, that is an autophagy substrate. Studies in mice and *Drosophila* [21,24^••^] showed that such inclusions did not form in p62-null animals when autophagy was compromised. One of the reasons that this occurs may be because p62 is a major constituent of such inclusions, possibly because it can oligomerise, and that it then serves as a seed for the generation of the ubiquitinated inclusions by sequestering ubiquitinated proteins onto its ubiquitin-binding domain [25^•^].

## p62 regulation, proteasome inhibition and tumourigenesis

If animals do not express p62, then the toxicity of autophagy compromise is also suppressed [24^••^]. One way that this may be mediated is because p62 appears to impact on the efficiency of the ubiquitin–proteasome system. When autophagy is compromised, p62 accumulates. This leads to a partial block in flux through the ubiquitin–proteasome system, but does not inhibit proteasome catalytic activity. P62 is both necessary and sufficient for this effect and appears to act by sequestering ubiquitinated substrates away from the normal proteins that mediate their delivery to the proteasome for degradation. When autophagy is inhibited, this leads to the accumulation of proteasome substrates (that are not autophagy substrates), like p53. The predicted effect of the accumulation of such short-lived cellular regulators is cell stress and increased susceptibility to toxic insults. Thus, a secondary compromise in the ubiquitin–proteasome system may underlie some of the toxic effects of autophagy deficiency [25^•^].

Modulation of p62 stability by autophagy has also been reported to play diverse roles in tumourigenesis [26,27^••^]. Jin *et al*. showed that p62 acts as a hub that recruits a ubiquitinated form of caspase 8 to p62 foci, allowing full activation of this caspase [26]. Mathew *et al*. reported that p62 accumulation in autophagy-incompetent cells leads to increased production of reactive oxygen species, which causes cellular perturbations like increased chromosomal instability, deregulated signal transduction and gene expression and increased predisposition to apoptosis in some cells. However, in solid tumours characterized by hypoxia, autophagy is upregulated, which promotes p62 degradation [28]. In this situation, downregulation of p62 increases Ras/ERK activity that enhances the survival and proliferation of cancer cells. However, this may be more complex, since p62 levels are often high in tumours, suggesting that the rate of p62 accumulation exceeds its degradation [29]. Indeed, p62 promotes lung adenocarcinoma formation by activated K-ras [29]. This, and the data of Matthew *et al.* [27^••^] suggest that p62 accumulation may promote oncogenesis. These new findings confirm the need for further investigation into the relationship between autophagy, p62 and tumourigenesis and tumour survival, since these may also be dependent on the cell type, tumour, tumour stage and environmental conditions.

## Cancers and p53 regulation of autophagy

**Figure 1:**
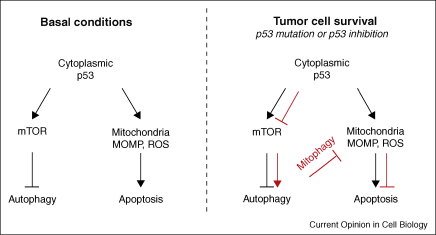
p53 regulation of autophagy and cell death. In basal conditions, cytoplasmic p53 inhibits autophagy via mTOR activation, and can induce apoptosis by increasing ROS (reactive oxygen species) production and by triggering MOMP (mitochondrial outer membrane permeabilization). In the context of tumour cell survival where p53 function is lost, either by mutation or by inhibition, mTOR is inhibited, thereby leading to autophagy induction. Enhancement of autophagy, including mitophagy, contributes to cell survival by inhibiting ROS and MOMP.

The principal tumour-suppressor protein, p53, transactivates a number of genes with a variety of functions including cell cycle arrest, apoptosis and metabolism. p53 is inactivated in approximately half of human cancers, either by mutations, or by increased expression of its inhibitors, which enhances tumour survival and development. Recent findings have demonstrated a role of p53 in autophagy regulation. Depending on p53 localisation (nuclear or cytoplasmic), autophagy is induced or inhibited, which contributes to cell survival or cell death responses. In basal conditions, Tasdemir *et al*. observed that cytoplasmic p53 inhibits autophagy by mTOR activation via AMP-dependent kinase [30^•^] (Figure 1). In p53-deficient cells (either by p53 mutation, inhibition or degradation), autophagy is restored and promotes cell survival, possibly via mitophagy (mitochondrial autophagy) [30^•^] (Figure 1). The cytoprotective effect of autophagy, while beneficial to cancer cells, is bad for the patient. Another relationship between p53 and autophagy was found by Bensaad *et al*., involving the phosphatase TIGAR, a p53-inducible protein [31,32]. TIGAR is involved in restraining levels of reactive oxygen species inside the cell, thereby inhibiting autophagy. When TIGAR function is inhibited, the increased levels of reactive oxygen species lead to autophagy induction [32].

## Mitophagy

**Figure 2:**
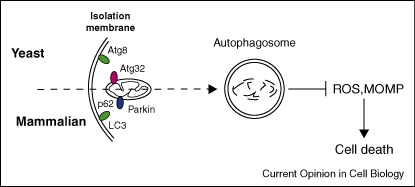
Cytoprotective effect of mitophagy. Direct interaction of the yeast Atg32 protein or indirect interaction of the mammalian Parkin protein with the autophagic protein LC3 leads to the degradation of mitochondria inside autophagosomes, in a process called mitophagy. Mitochondrial degradation protects cell against apoptosis by preventing ROS (reactive oxygen species) formation and MOMP (mitochondrial outer membrane permeabilization).

Mitochondria play an important role in cell homeostasis and cell survival by regulating energy production (oxidative phosphorylation) and apoptosis. Consequently, cells need to be able to monitor mitochondrial quality and quantity. The major route for mitochondrial degradation is via mitophagy. However, the molecular mechanisms for mitochondria's recognition by autophagy (either selective, random or both) remain to be clarified in mammalian systems. Recently, an important contribution to our understanding of mitophagy in yeast has been reported by the discovery of a mitophagy receptor, Atg32 [33^••^,34^••^] (Figure 2). Importantly, Atg32 is involved only in mitophagy and not in bulk autophagy or the Cvt pathway [33^••^,34^••^]. These two studies indicate that mitophagy is indeed a selective process. However, additional work is required in order to understand the physiological significance of mitophagy, since no defect in mitochondrial activity has been found in *Atg32* knockout cells. Although an Atg32 homologue has not been found in mammalian cells, mitophagy is believed to be selective to some extent because it employs the ubiquitin ligase, Parkin (mutated in rare autosomal recessive forms of Parkinson's disease), which is translocated from the cytosol to dysfunctional mitochondria to promote selective degradation [35^•^]. Parkin may be acting though the ubiquitination of mitochondrial proteins. Such ubiquitinated proteins may serve as binding sites for p62, which can then target the mitochondria to autophagosomes via its interaction with LC3. In neurodegenerative disorders, such as Parkinson's disease (PD), in which the accumulation of aggregated/misfolded proteins and the dysfunction of mitochondria are the two major defects, mitophagy appears to be cytoprotective [36] (Figure 2). There may be other molecules that target mitochondria for autophagic clearance in mammals, since the expression of Parkin is limited. One candidate is Nix (also known as Bnip3L), which is important for mitochondrial removal in erythroid maturation, and may also play a role in mitophagy in other tissues [37].

## Immune response

**Figure 3:**
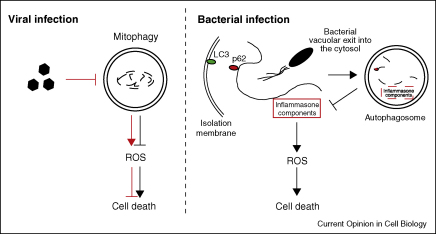
Cytoprotective effect of autophagy in the context of infectious diseases. In cases of viral infection that lead to autophagy inhibition (red arrows), mitophagy defects contribute to ROS (reactive oxygen species) production and thereby trigger cell death. In cases of bacterial infection, where bacteria can escape the vacuolar environment following vacuolar lysis, membrane remnants are targeted by autophagy for degradation in a p62-dependent manner. Degradation of these membrane remnants, where specific inflammasome components are recruited, contributes to cell survival by preventing ROS formation.

Autophagy has been demonstrated to play a role in the clearance of pathogens like viruses and bacteria [38^•^,39]. However, in some cases, autophagosomes serve as a reservoir for pathogen replication [38^•^,40]. In the study of Alirezaei *et al*., simian immunodeficiency virus (SIV)-infected microglia have impaired autophagy, resulting in decreased neuronal survival [41]. Also, in HIV-infected individuals, autophagy inhibition may contribute to neurotoxicity [41] (Figure 3). Indeed, in the same study, rapamycin treatment prevented neurodegenerative processes by restoring autophagy [41]. Recent work has implicated autophagy for membrane remnant clearance following bacterial vacuolar exit into the cytosol [42^•^]. The degradation of these membrane remnants by autophagy is a p62-dependent process and contributes to maintain homeostasis, as inflammatory response components are specifically recruited on these structures [42^•^] (Figure 3).

## Conclusion

**Figure 4:**
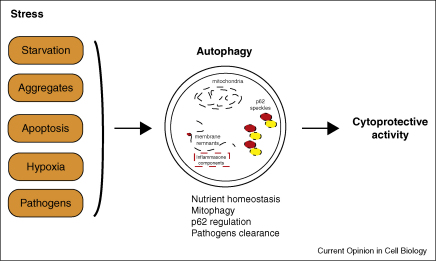
Cytoprotective effect of autophagy. In stress conditions, autophagy exerts its prosurvival roles by promoting nutrient and bioenergetic homeostasis, aggregate clearance, p62 regulation, mitophagy (for mitochondria-dependent apoptosis), and the removal of intracellular pathogens.

In this review, we have covered some of the contexts where autophagy may be cytoprotective (Figure 4). The relationship between autophagy and cell death is complex and this is, in part, influenced by the emerging data showing coregulation of autophagy and apoptosis. For example, Bcl-2 and Bcl-xl inhibit both apoptosis and autophagy [43^••^]. Also, the autophagy gene *Atg5* has been reported to induce apoptosis when overexpressed [44]. While autophagy generally protects against apoptosis, recent data suggest that caspase activation during apoptosis inhibits autophagy because of cleavage and inactivation of the autophagy protein Beclin-1 [45^•^]. Thus, the interplay between autophagy and apoptosis and other forms of cell death is a field ripe for further exploration.

## References and recommended reading

Papers of particular interest, published within the period of review, have been highlighted as:

• of special interest
•• of outstanding interest
